# Public Perception of Physicians Who Use Artificial Intelligence

**DOI:** 10.1001/jamanetworkopen.2025.21643

**Published:** 2025-07-17

**Authors:** Moritz Reis, Florian Reis, Wilfried Kunde

**Affiliations:** 1 Institute of Psychology, University of Wuerzburg, Wuerzburg, Germany; 2 Judge Business School, University of Cambridge, Cambridge, United Kingdom; 3 Institute of Medical Informatics, Charité–Universitätsmedizin Berlin, Berlin, Germany

## Abstract

This survey study evaluates how perceptions of physicians’ competence, trustworthiness, and empathy vary based on physician use or nonuse of artificial intelligence (AI).

## Introduction

Artificial intelligence (AI) is rapidly gaining importance in medicine.^1^ Recent findings, however, indicate potential concerns from the patients’ and the public’s perspective.^2^ So far, such research focused on attitudes toward medical AI tools^3^ and AI-generated medical advice.^4^ In contrast, little is known about the public perception of physicians themselves who use AI. This online study explored how statements on different types of AI use (diagnostic, therapeutic, and administrative) influence the public’s perception of respective physicians.

## Method

This survey study was registered prior to data collection, which took place in January 2025. We recruited an online quota sample of US adults based on the 2021 census via Prolific. Participants were shown fictitious advertisements for family doctors that might be encountered on social media or billboards (eFigure 1 in Supplement 1). Participants were randomized into 4 groups. All participants received similar advertisements, only differing in one crucial aspect. That is, we varied between groups whether the advertisement made no statement on AI use (control condition) or mentioned that the respective physician utilizes AI for administrative, diagnostic, or therapeutic purposes (eFigure 2 in Supplement 1). Participants rated the presented physician regarding perceived competence, trustworthiness, and empathy as well as their willingness to make an appointment with the physician on a 5-point scale. For each dimension, we compared ratings between all 4 conditions with 2-sided *t* tests. Bonferroni-Holm correction was used to adjust the significance level based on the number of rating dimensions (baseline significance was defined as 2-sided *P* < .05). We followed the AAPOR reporting guideline, and R version 4.1.1 (R Project for Statistical Computing) was used for analyses. This study was approved by the ethics committee of the Institute of Psychology of the University of Wuerzburg, and all participants provided written informed consent. The eMethods in Supplement 1 provides additional details on the sample and procedure.

## Results

Participants included 1276 adults (680 [53.3%] women, 584 [45.8%] men, 7 [0.5%] nonbinary individuals, and 5 participants [0.4%] who preferred to not disclose their gender; mean [SD] age, 46.2 [15.6] years). In every AI condition, the portrayed physician was perceived as significantly less competent (control: 3.85 [95% CI, 3.75-3.94] points; administrative AI: 3.71 [95% CI, 3.61-3.80] points; diagnostic AI: 3.66 [95% CI, 3.56-3.76] points; therapeutic AI: 3.58 [95% CI, 3.48-3.68] points), less trustworthy (control: 3.88 [95% CI, 3.79-3.96] points; administrative AI: 3.66 [95% CI, 3.57-3.75] points; diagnostic AI: 3.62 [95% CI, 3.52-3.72] points; therapeutic AI: 3.61 [95% CI, 3.50-3.71] points), and less empathic (control: 4.00 [95% CI, 3.92-4.09] points; administrative AI: 3.80 [95% CI, 3.71-3.88] points; diagnostic AI: 3.82 [95% CI, 3.73-3.92] points; therapeutic AI: 3.72 [95% CI, 3.62-3.82] points) compared with the control condition (Table and Figure). Moreover, participants indicated a significantly lower willingness to make an appointment with the portrayed physician, if any type of AI use was mentioned (control: 3.61 [95% CI, 3.50-3.73] points; administrative AI: 3.32 [95% CI, 3.21-3.44] points; diagnostic AI: 3.16 [95% CI, 3.03-3.30] points; therapeutic AI: 3.15 [95% CI, 3.01-3.29] points). There was no significant difference between the AI conditions for any rating dimension.

**Table.** Comparison of Scores Across Conditions

Group	Score, mean (95% CI)	vs Admin AI		vs Diagnostic AI		vs Therapeutic AI	
		*P* value	Cohen *d*	*P* value	Cohen *d*	*P* value	Cohen *d*
Competence							
Control	3.85 (3.75-3.94)	.04	0.16	.007	0.22	<.001	0.30
Admin AI	3.71 (3.61-3.80)	NA	NA	.51	0.05	.08	0.14
Diagnostic AI	3.66 (3.56-3.76)	NA	NA	NA	NA	.28	0.09
Therapeutic AI	3.58 (3.48-3.68)	NA	NA	NA	NA	NA	NA
Trustworthiness							
Control	3.88 (3.79-3.96)	.001	0.26	<.001	0.30	<.001	0.32
Admin AI	3.66 (3.57-3.75)	NA	NA	.53	0.05	.42	0.06
Diagnostic AI	3.62 (3.52-3.72)	NA	NA	NA	NA	.86	0.01
Therapeutic AI	3.61 (3.50-3.71)	NA	NA	NA	NA	NA	NA
Empathy							
Control	4.00 (3.92-4.09)	.001	0.28	.005	0.22	<.001	0.34
Admin AI	3.80 (3.71-3.88)	NA	NA	.65	0.04	.23	0.09
Diagnostic AI	3.82 (3.73-3.92)	NA	NA	NA	NA	.13	0.12
Therapeutic AI	3.72 (3.62-3.82)	NA	NA	NA	NA	NA	NA
Willingness to make appointment							
Control	3.61 (3.50-3.73)	<.001	0.28	<.001	0.40	<.001	0.41
Admin AI	3.32 (3.21-3.44)	NA	NA	.08	0.14	.06	0.15
Diagnostic AI	3.16 (3.03-3.30)	NA	NA	NA	NA	.87	0.01
Therapeutic AI	3.15 (3.01-3.29)	NA	NA	NA	NA	NA	NA

Abbreviations: Admin, administrative; AI, artificial intelligence; NA, not applicable.

**Figure 1.**
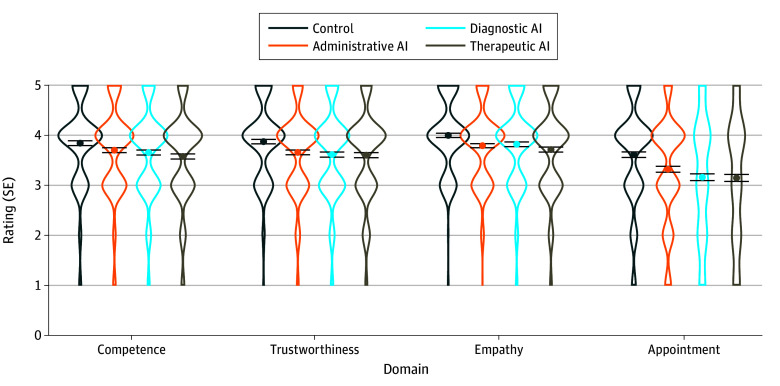
Mean Ratings for Each Experimental Condition and Rating Dimension Ratings are scaled from 1 to 5. Error bars show SEs of the individual means.

## Discussion

In line with prior research,^2,3,4^ our results indicate that the public has certain reservations about the integration of AI in health care. While the present effect sizes are relatively small, in particular regarding AI use for administrative purposes, they may be highly relevant as trust in health care practitioners is closely linked to subjective treatment outcomes.^5^ Potential reasons for existing skepticism may include concerns that physicians rely too much on AI and that the use of AI could reduce patient-physician interactions as well as concerns about data protection and rising health care costs.^6^ From the physician’s perspective it thus may be important to transparently communicate the rationale for using AI and to emphasize its potential benefits for the patient. Limitations to the generalizability of our results are the use of hypothetical scenarios, the somewhat artificial nature of our stimuli, and the recruitment of a sample that agreed to participate in such experiments. Future research should extend these findings to even more realistic settings and explore potential moderating factors, such as patients’ experience with AI and with digital tools in general.

## Notes

Supplement 1. **eFigure 1.** Stimuli for Each Experimental Condition **eFigure 2.** Experiment Setup With Experimental Conditions and Rating Dimensions
eMethods.

eReferences.

Supplement 2.
Data Sharing Statement
