# Supplementary material for: Public Perception of Physicians Who Use Artificial Intelligence
Source: JAMA Netw Open. 2025 Jul 17;8(7):e2521643. doi: 10.1001/jamanetworkopen.2025.21643 (PMC12272287; doi:10.1001/jamanetworkopen.2025.21643)
Supplement: Supplement 1. — eFigure 1. Stimuli for Each Experimental Condition eFigure 2. Experiment Setup With Experimental Conditions and Rating Dimensions eMethods. eReferences. [file jamanetwopen-e2521643-s001.pdf]

Supplemental Online Content

Reis M, Reis F, Kunde W. Public Perception of physicians who use artificial intelligence. *JAMA Netw Open.* 2025;8(7):e2521643. doi:10.1001/jamanetworkopen.2025.21643

eFigure 1. Stimuli for Each Experimental Condition

eFigure 2. Experiment Setup With Experimental Conditions and Rating Dimensions

eMethods.

eReferences.

This supplemental material has been provided by the authors to give readers additional information about their work.

© 2025 Reis M et al. *JAMA Network Open*.

eFigure 1. Stimuli for Each Experimental Condition

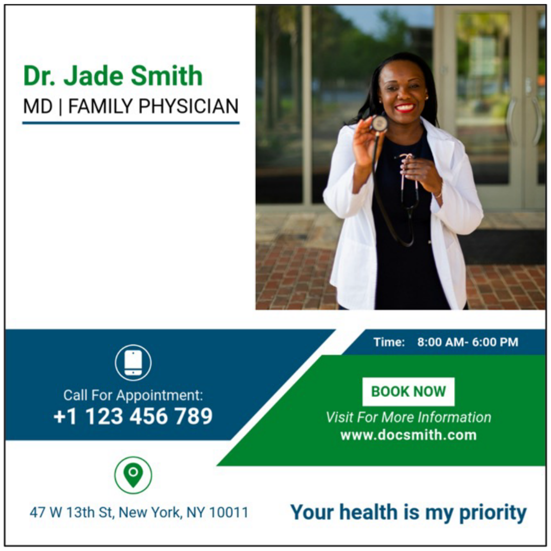

Control condition

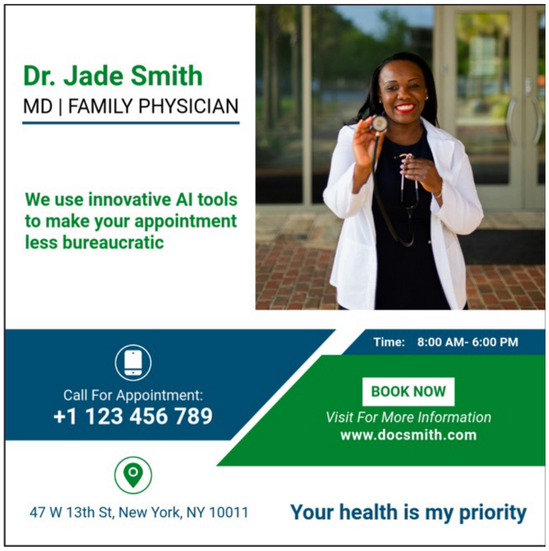

Administrative AI

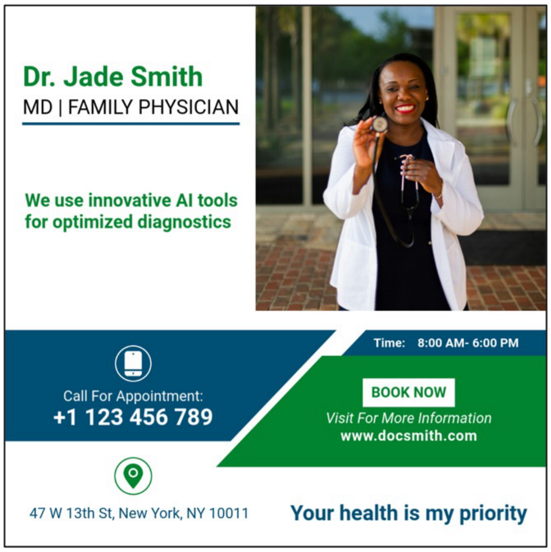

Diagnostic AI

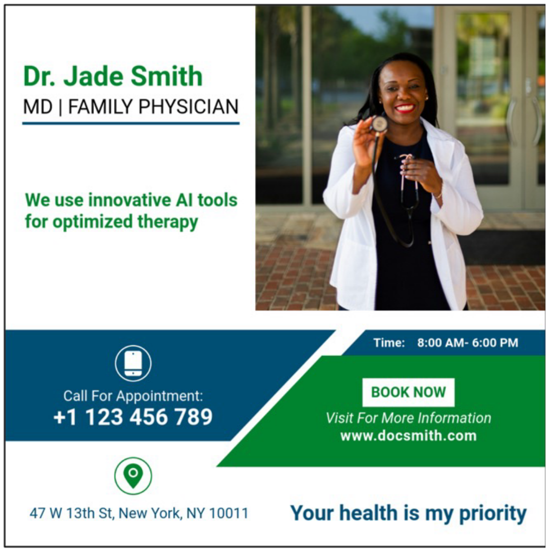

Therapeutic AI

© 2025 Reis M et al. *JAMA Network Open*.

eFigure 2. Experiment Setup With Experimental Conditions and Rating Dimensions

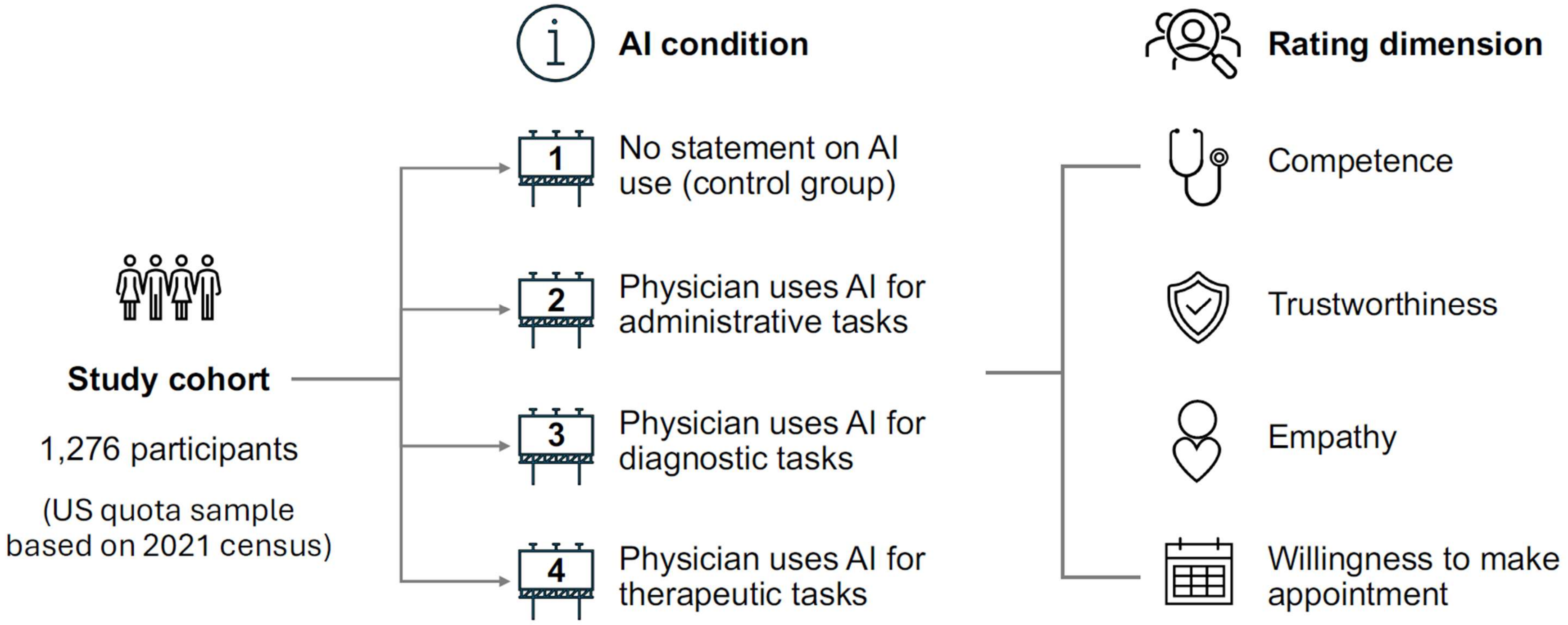

A cohort of 1276 participants was randomly allocated to four conditions. In each condition, advertisements with different statements regarding the use of AI were presented. Afterwards, each participant rated the shown physician regarding all dimensions.

© 2025 Reis M et al. *JAMA Network Open*.

eMethods

This study was pre-registered prior to data collection (https://osf.io/snmh2). The sample was recruited via Prolific (http://www.prolific.com).

Sample

We based our sample size on a formal power analysis. That is, we aimed for a statistical power of $1\text{-}\beta \geq 90\%$ to detect an effect size of $d = 0.3$ ($\alpha = .0125$, 2-sided testing; calculated with the *power.t.test* function in the *statistics* package of R version 4.1.1). This effect size estimate is based on prior research on public attitudes toward AI in healthcare (e.g., $0.21 \leq d \leq 0.46$ for (1)). If participants did not finish the entire study, their dataset was replaced by a new one ($n = 155$). Moreover, if participants did not provide the correct response to the attention check at the end of the experiment (see Experimental procedure for details), their dataset was also replaced by a new one ($n = 333$).

The aimed sample size was stratified across three demographics: age, gender and ethnicity (based on the US census 2021, done via the "representative sample" feature of Prolific). There were five brackets for age (18-24, 25-34, 35-44, 45-54 and 55+) and ethnicity (Asian, Black, Mixed, White and Other). Cross-stratified subgroups were filled on a first-come first-serve basis. More than half of the sample indicated a university degree as their highest level of education (3 no formal qualification, 13 secondary education, 481 high school, 492 bachelor, 223 master, 49 PhD, 15 prefer not to say). 674 participants (52.8%) described themselves as a patient and 104 participants (8.2%) indicated that they work in a healthcare-related profession.

Participants indicated a diverse range of private and work-related AI-use frequencies ("never": $n = 204$, "less than once a month": $n = 297$, "at least once a month but not every week": $n = 298$, "at least once a week": $n = 328$, "at least once a day": $n = 149$). When asked whether the increasing integration of AI into healthcare comes with more risks or opportunities, 326 participants opted for "more risks than opportunities", 364 participants opted for "more opportunities than risks" and 586 participants opted for "equally balanced risks and opportunities". Most of the participants agree that AI will have a remarkable impact on healthcare in the future ("no impact": $n = 18$, "minor impact": $n = 82$, "moderate": $n = 344$, "significant": $n = 550$, "highly significant": $n = 282$). The total study data was collected between the 21st and the 22nd of January 2025. Each participant received £0.50 for participation. We preregistered the analysis plan, our hypotheses and the targeted sample size before data collection had started (https://osf.io/snmh2; please note that contrary to our preregistration, we do not report additional mixed effect regressions, because of the single shot design of the study and an according lack of within-subject variance).

Experimental procedure

After providing informed consent, participants were instructed to take a close look at a fictitious advertisement for a family doctor (see eFigure 1). To ensure equally large group sizes, they were assigned to the different conditions via block-randomization. Participants were asked to evaluate the presented physician on four dimensions (perceived competence, trustworthiness, empathy and willingness to make an appointment). Participants indicated their responses on 5-point Likert scales, going from 'very incompetent' to 'very competent', from 'very untrustworthy' to 'very trustworthy', from 'very unemphatic' to 'very emphatic' and from 'very unlikely' to 'very likely', respectively. Afterwards, we queried participants' attitudes toward AI. That is, we assessed their frequency of using AI-based tools (response options: never, rarely, occasionally, frequently, very frequently), asked whether they perceive the increasing integration of AI in healthcare as coming with more risks or opportunities (response options: more risks, neutral, more opportunities) and queried their view on the impact of AI on healthcare (response options: no, minor, moderate, significant, highly significant). Next, we collected demographic information (gender, age, educational level), assessed participants' patient status ('Based on your current health status, would you describe yourself as a patient?'; response options: yes, no, prefer not to say) and asked whether they received a healthcare-related training or work in a healthcare-related profession ('Based on your training or current profession, would you describe yourself as a healthcare professional?'; response options: yes, no, prefer not to say). Participants could also indicate their exact profession, if the latter question was answered with 'yes'. As an attention check, at the very end of the study, participants should select the presented statement on AI use ('There was no statement on AI tools in the advertisement', 'Innovative AI tools to make your appointment less bureaucratic', 'Innovative AI tools for optimized diagnostics', 'Innovative AI tools for optimized therapy'). Finally, we debriefed participants about the study purpose.

eReferences

1. Reis M, Reis F, Kunde W. Influence of believed AI involvement on the perception of digital medical advice. *Nat Med*. 2024;30(11):3098-3100. doi:10.1038/s41591-024-03180-7

© 2025 Reis M et al. *JAMA Network Open*.
