# Supplementary material for: Public Perception of Physicians Who Use Artificial Intelligence
Source: JAMA Netw Open. 2025 Jul 17;8(7):e2521643. doi: 10.1001/jamanetworkopen.2025.21643 (PMC12272287; doi:10.1001/jamanetworkopen.2025.21643)
Supplement: Supplement 2. — Data Sharing Statement [file jamanetwopen-e2521643-s002.pdf]

Data Sharing Statement

Reis. Public Perception of Physicians Who Use Artificial Intelligence. *JAMA Netw Open*. Published July 17, 2025. doi:10.1001/jamanetworkopen.2025.21643

Data
Data available: Yes
Data types: Deidentified participant data
How to access data: Data is available at https://osf.io/fgcra/
When available: With publication

Supporting Documents
Document types: Statistical/analytic code
How to access documents: Analysis code is available at https://osf.io/fgcra/
When available: With publication

Additional Information
Who can access the data: Data will be available to anyone via a link in the published manuscript.
Types of analyses: Data will be available for any purpose.
Mechanisms of data availability: Data will be available to anyone via a link in the published manuscript.
